# Urinary Congophilia Confirmed With the CapCord Test Is Associated With Pregnancy Outcomes in Women With Early-Onset Pre-eclampsia

**DOI:** 10.3389/fmed.2021.700157

**Published:** 2021-08-02

**Authors:** Benshuo Cai, Xiaoying Yuan, Xingmin Li, Jun Xu, Juan Du

**Affiliations:** ^1^Department of Obstetrics and Gynecology, Shengjing Hospital of China Medical University, Shenyang, China; ^2^Shuwen Biotech Company Ltd., Deqing, China

**Keywords:** congophilia, misfolded protein, early-onset pre-eclampsia, late-onset pre-eclampsia, pregnancy outcome

## Abstract

**Background:** The association between misfolded proteins presented in the urine of pregnant women and pregnancy outcomes associated with early-onset pre-eclampsia (PE) remains unclear. This study aimed to investigate this association to examine the predictive value of urinary congophilia in the prognostication of pregnancy outcomes in this patient group in the Chinese population.

**Materials and Methods:** This study included 1,397 patients, of which 46, 147, and 8 patients had gestational hypertension, PE, and chronic hypertension, respectively, and 1,196 were healthy controls undergoing the CapCord test for urinary congophilia. Patients with PE were divided into early- and late-onset groups. Patients with early-onset PE were further divided into iatrogenic prematurity and full-term delivery groups, the rates of urinary congophilia were compared between the groups; additionally, this patient group was divided into positive and negative urinary congophilia groups, clinical characteristics and pregnancy outcomes were compared between the groups. Univariate and multivariate logistic regression analyses were performed.

**Results:** A total of 113 (76.9%) of 147 patients in the PE group had urinary congophilia; this rate was higher than that observed in the other three groups (χ^2^ = 780.892, *p* < 0.001). Gestational age in the early-onset PE group at both onset and delivery was lower than that in the late-onset PE group (*p* < 0.001). The rates of iatrogenic prematurity and hemolysis, elevated liver enzymes, and low platelet count syndrome were both higher in the early-onset PE group than in the late-onset PE group (*p* < 0.001, *p* < 0.05). In addition, the rate of urinary congophilia in the early-onset PE group was higher than that in the late-onset PE group (χ^2^ = 13.297, *p* < 0.001). Urinary congophilia was an independent risk factor for iatrogenic prematurity among patients with early-onset PE in both univariate [odds ratio (OR) 17.143, 95% confidence interval (CI): 4.719–62.271; *p* < 0.001] and multivariate (OR 18.174; 95% CI: 4.460–74.063; *p* < 0.001) analyses. Patients with early-onset PE and urinary congophilia were more likely than their counterparts without urinary congophilia to deliver at a lower gestational age, present with iatrogenic prematurity, and have a shorter latency period between onset and delivery.

**Conclusion:** Urinary congophilia confirmed with the CapCord test may help predict pregnancy outcomes in patients with early-onset PE.

## Introduction

Pre-eclampsia (PE) is the leading contributor to maternal and fetal mortality worldwide (1). It is defined as new-onset hypertension and proteinuria after 20 weeks of gestation (2), accounting for 17–24% of maternal deaths in low-income settings (3). Untreated PE may lead to eclampsia, renal damage, cerebrovascular accidents, microangiopathic hemolytic anemia, liver failure, and pulmonary edema, all of which increase the risk of maternal death (4, 5). Delivery is the only effective treatment for PE; however, preventing stillbirth and iatrogenic prematurity remains a challenge. Depending on the gestational age at onset, PE is classified as early- (or placental) or late-onset (or maternal) (6, 7); these subtypes appear to have different etiologies. Specifically, early-onset PE is associated with abnormal placentation; by contrast, late-onset PE is associated with an interaction between a presumably normal placenta and maternal factors such as endothelial dysfunction and microvascular damage (8, 9). The pathogenesis of PE is yet to be elucidated; however, some evidence suggests that uteroplacental hypoperfusion may lead to PE (10, 11). Meanwhile, impaired spiral artery remodeling in the uterus, which may lead to the release of antiangiogenic factors from the ischemic placenta into the maternal circulation, is a two-stage model considered central to research in the pathogenesis of PE (12).

In human cells, linear amino acid multimers can be converted into functional proteins with three-dimensional structures able to perform their function. Some diseases are associated with protein folding disturbance, which results in the formation of misfolded proteins, such as in Alzheimer's and Parkinson's diseases, and prion diseases (13–18). Recent studies have shown that misfolded proteins may accumulate in the urine, serum, and placenta of patients with PE (19–24). Urinary misfolded proteins can be detected by a point-of-care urinary Congo red test (25). Congo red is a kind of synthetic diazo dye with specific affinity for misfolded proteins (26–28). The affinity of misfolded proteins to Congo red is known as congophilia (29, 30). Buhimschi et al. proposed that urine samples of women with PE exhibited congophilia; additionally, the rate of urinary congophilia was higher among women with severe PE with indications for delivery than among their counterparts that were either healthy or diagnosed with chronic or gestational hypertension (21). However, the origin of misfolded proteins in the urine of women with PE remains unclear. Placental hypoxia and ischemia resulting from impaired placentation in PE may lead to endoplasmic reticulum stress (ERS) in the placenta (31–33). ERS may lead to chronic activation of unfolded protein response (UPR) pathways (34–36), which aim to restore endoplasmic reticulum homeostasis by removing the misfolded proteins. The activation of placental UPR occurs in early- but not in late-onset PE or normotensive controls (33). Based on this evidence, we hypothesized that the placenta may be the main source of the misfolded proteins in the urine of patients with early-onset PE and the presence of misfolded proteins in the urine may be linked to the pathogenesis of early-onset PE but not late-onset PE. In this study, we, for the first time, compared the urinary congophilia of patients with different types of PE and normotensive controls in the Chinese population to investigate the association between the presence of misfolded proteins in the urine and early-onset PE, as well as the possible origin of the misfolded proteins in the urine in the Chinese population. Furthermore, we examined the association of urinary congophilia with the pregnancy outcomes in Chinese patients with early-onset PE to assess the predictive value of urinary congophilia in the prognostication of pregnancy outcomes in this patient group.

## Materials and Methods

### Study Design

The protocol of this study was approved by the Institutional Review Board of Shengjing Hospital of China Medical University, Shenyang, Liaoning Province, China (Approval number: 2018PS195K). The need for obtaining informed consent was waived owing to the use of residual urine samples and the minimal risks involved. The study was conducted according to the principles expressed in the Helsinki Declaration.

Pregnant women aged ≥18 years and at the gestational age of ≥20 weeks, admitted to our hospital between May 2017 and August 2018 were eligible for this study. Included patients were divided into gestational hypertensive, PE, chronic hypertensive, and normotensive groups. The PE group was further subdivided into early- and late-onset groups. The patients' clinical characteristics and rates of urinary congophilia were compared among the groups. Pregnancy outcomes of the patients with early-onset PE were categorized as iatrogenic prematurity and full-term delivery. Patients with early-onset PE were further subdivided into positive and negative urinary congophilia groups.

### Diagnostic Criteria

Hypertensive disorders of pregnancy were determined according to the 2018 International Society for the Study of Hypertension in Pregnancy Classification, Diagnosis, and Management Recommendations for International Practice (2).

Women were excluded from the present study if they were diagnosed with any of the followings: diabetes mellitus, respiratory disease, blood system disease, liver disease, renal disease, heart disease, fetal genetic and congenital malformation, abortion or fetal death, twin or multiple pregnancy, history of assisted reproductive technology use, infections or pro-inflammatory states, autoimmune disease, or cancer. Women with incomplete clinical information were also excluded. Early- and late-onset PE were defined by the gestational age at onset of disease, <34 and ≥ 34 weeks, respectively (6).

### Urine Sample and Clinical Data Collection

Midstream urine samples were collected from all patients for the assessment of congophilia. In patients with hypertensive disorders of pregnancy, urine samples were collected at the time of disease onset. All patients with hypertensive disorders of pregnancy were followed-up until post-delivery. Data on maternal age, gestational age at PE onset and delivery, pregnancy outcomes, and the presence of the hemolysis, elevated liver enzymes, and low platelet count syndrome (HELLP syndrome) were collected. Investigators were blinded to any personal data during congophilia measurement.

### Detection of Misfolded Proteins in Urine

**Figure 1:**
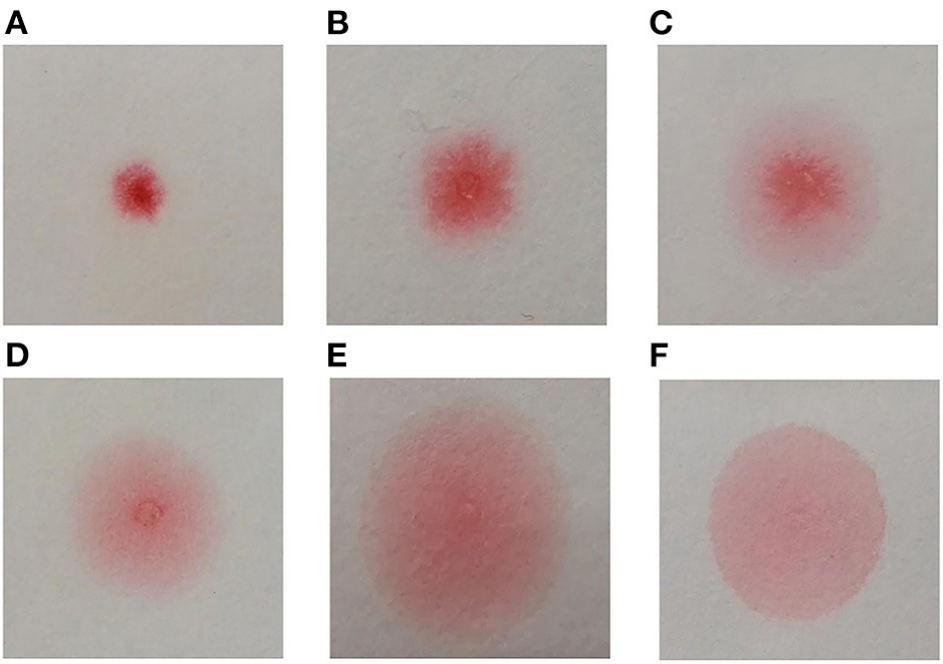
Congo red bound to misfolded proteins in an aqueous solution migrates differentially on cellulose membrane, forming different dyeing patterns. The differences are especially apparent when the solution is slowly released into small area on the cellulose membrane through a fine-tipped capillary tube. The more Congo red is bound to misfolded proteins, the dye spreads more evenly on the membrane. The device we used included a plastic pipette to drop urine to a well-containing Congo red (0.1 mg/ml), and a capillary applicator to transfer the mixture to cellulose membrane compartment and slowly released. The test produces a result within 3 min. Classification of Congo red staining patterns **(A)** Small non-diffused red dot; **(B)** Mildly diffused dot, scarlet pseudopodium; **(C)** Diffused dot, scarlet pseudopodium, pink penumbra; **(D)** Small dot, irregular partly diffused pale red penumbra; **(E)** Red and scarlet dot, partly diffused pale red penumbra; **(F)** Large uniform pale diffused dot. The patterns **(D–F)** were classified as “positive” and patterns **(A–C)** as “negative.”

A point-of-care device employing the capillary tube-based slow release method (the CapCord test, available from Shuwen Biotech. Zhejiang, China) was used to detect misfolded proteins in urine samples (25). Each scorer classified the pattern of the dye into six categories relative to the reference pattern, based on the evenness of the spread and tendency of the dye to concentrate in a limited central area (Figure 1). All scorers were trained to ensure the consistency of approach.

### Statistical Methods

Clinical characteristics and pregnancy outcomes were compared among the groups, using the analysis of variance or Mann–Whitney *U*-test. The difference in counts among the groups was assessed with the chi-squared test or Fisher's exact probability test. Univariate and multivariate logistic regression analyses were performed. Survival curves were compared using the Kaplan–Meier method and the log-rank test. *P*-values < 0.05 were indicative of significant findings. All statistical analyses were performed using the Statistical Product and Service Solutions (SPSS) software ver. 23.0 (IBM Corp., Armonk, NY, USA).

## Results

This study included 1,397 patients. The CapCord test was performed for all patients. Table 1 presents the incidence of urinary congophilia in four groups. The differences of the rates of positive urinary congophilia among different groups were significant.

**Table 1:** Incidence of positive urinary congophilia in four groups.

**Variables**	**Gestational hypertensive (*n* = 46)**	**Pre-eclampsia (*n* = 147)**	**Chronic hypertensive (*n* = 8)**	**Normotensive (*n* = 1,196)**	*****χ^2^*****	***P-*value**
Rate of positive urinary congophilia	1 (2.2%)	113 (76.9%) ^*^	0 (0%)	31 (2.6%)	780.892	<0.001

*Values are n and n/N (%)*

* *p < 0.001 vs. gestational hypertensive, chronic hypertensive, and normotensive (chi-squared test or Fisher exact probability test)*.

Table 2 presents comparisons of the clinical characteristics and rates of urinary congophilia between early- and late-onset PE groups. The gestational age of the early-onset PE group at both PE onset and delivery was significantly lower than that of the late-onset PE group. The rates of iatrogenic prematurity and HELLP syndrome were both significantly higher in the early- than in the late-onset PE group. In addition, the rate of urinary congophilia in the early-onset PE group was significantly different from that in the late-onset PE group.

**Table 2:** Clinical characteristics and incidence of positive urinary congophilia in early- and late-onset pre-eclampsia groups.

**Variables**	**Early-onset (*n* = 102)**	**Late-onset (*n* = 45)**	***Z***	*****χ^2^*****	***P-*value**
Gestational age at onset	28.8 (26, 31.3)	37 (35.3, 38.2)	−9.653	/	<0.001
Gestational age at delivery	32.6 (30.1, 35.4)	38.1 (36.8, 38.6)	−7.584	/	<0.001
Iatrogenic prematurity	86 (84.3%)	11 (24.4%)	/	49.865	<0.001
HELLP syndrome	18 (17.6%)	1 (2.2%)	/	6.601	<0.05
CapCord test-positive	87 (85.3%)	26 (57.8%)		13.297	<0.001

*Values are median (P25, P75), n or n/N (%). HELLP syndrome, hemolysis, elevated liver enzymes, and low platelet count syndrome*.

Urinary congophilia was an independent risk factor for iatrogenic prematurity in both univariate [odds ratio (OR) 17.143; 95% confidence interval (CI) 4.719–62.271; *p* = 0.000] (Table 3) and multivariate (OR 18.174; 95% CI 4.460–74.063; *p* = 0.000) analyses.

**Table 3:** Clinical characteristics of patients with early-onset PE and iatrogenic prematurity or full-term delivery.

**Variables**	**Iatrogenic prematurity (*n* = 86)**	**Full-term (*n* = 16)**	**Univariate analysis**	
			**OR (95%CI)**	***P*-value**
Age (years)	30.8 ± 5.3	31.1 ± 4.5	0.989 (0.891, 1.098)	0.842
Gestational age at onset	28.1 ± 3.9	26.8 ± 5.9	1.069 (0.948, 1.205)	0.279
HELLP	17 (19.8%)	1 (6.3%)	3.696 (0.456, 29.958)	0.221
Rate of positive urinary congophilia	80/86 (93.0%)	7/16 (43.8%)	17.143 (4.719, 62.271)	<0.001

*Values are mean ± standard deviation, n or n/N (%)*.

A significant difference was found in the gestational age at delivery between patients with and without urinary congophilia in the early-onset PE group. The positive urinary congophilia group presented with a shorter latency period between onset and delivery than did the negative urinary congophilia group; finally, a significant difference was observed in the rate of iatrogenic prematurity between the groups with and without urinary congophilia within the early-onset PE group (Table 4).

**Table 4:** Clinical characteristics and pregnancy outcomes among patients with positive and negative urinary congophilia and early-onset pre-eclampsia.

**Variables**	**Positive urinary congophilia (*n* = 87)**	**Negative urinary congophilia (*n* = 15)**	***F***	***Z***	*****χ^2^*****	***P*-value**
Age (years)	30.7 ± 5.3	31.9 ± 3.6	0.635		/	0.428
Gestational age at onset (weeks)	28.1 ± 4.2	26.8 ± 4.8	1.167		/	0.283
Gestational age at delivery (weeks)	32.1 ± 3.4	36.2 ± 3.1	19.529		/	<0.001
Latency between onset and delivery (days)	19 (9, 38)	52 (33, 119)	/	−3.502	/	<0.001
HELLP syndrome (%)	17/87 (19.5%)	1/15 (6.7%)	/	/	0.708	0.400
Iatrogenic prematurity (%)	80/87 (92.0%)	6/15 (40%)	/	/	26.111	<0.001

*Values are mean ± standard deviation, median (P25, P75), n or n/N (%)*.

**Figure 2:**
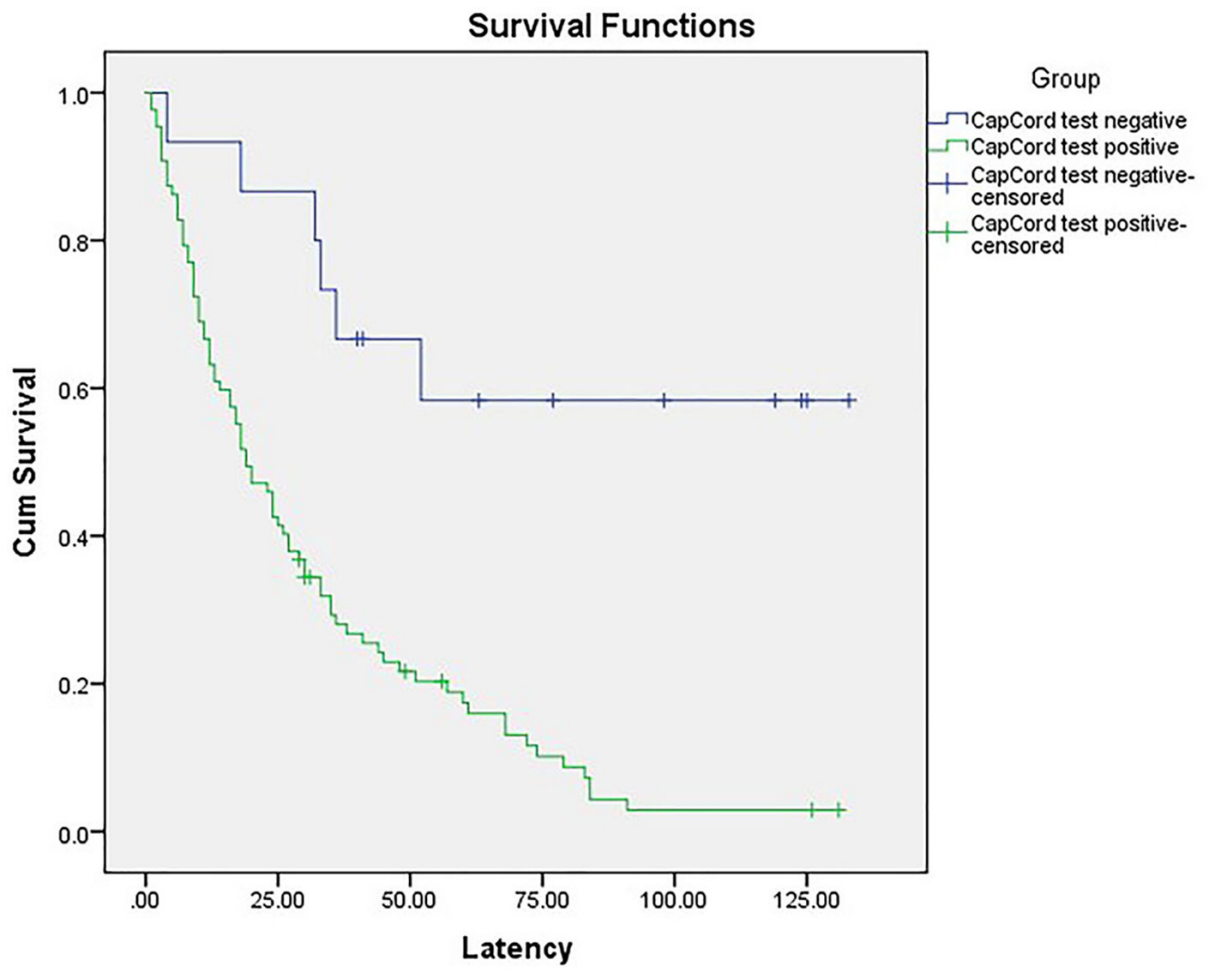
The Kaplan-Meier survival curves showed higher rates of iatrogenic prematurity among patients with urinary congophilia than among their counterparts without this condition (χ^2^ = 15.976, *p* < 0.001).

In total, 86 patients with iatrogenic prematurity comprised the early-onset PE group. The Kaplan-Meier survival curves showed higher rates of iatrogenic prematurity among patients with urinary congophilia than among their counterparts without this condition (χ^2^ = 15.976, *p* < 0.001) (Figure 2). These findings suggest that pregnancy outcomes are poorer in early-onset PE patients with urinary congophilia than in their counterparts without this condition.

## Discussion

Previous studies on the presence of misfolded proteins in PE patients mostly involved Western populations (19–21, 37–39). The present study confirms that urinary congophilia occurs in Chinese women with PE. The present findings also support those previously reported by Buhimschi, wherein women with PE were more likely to have urinary congophilia than their counterparts with gestational hypertension, chronic hypertension, or normotension (37). Recent studies have shown that ischemia, hypoxia, and production of pro-inflammatory cytokines, all of which are associated with PE, can lead to protein misfolding (40) and initiate ERS (41, 42), which results in the chronic activation of UPRs. Yung et al. have shown that activation levels of placental UPR in patients with early-onset PE are significantly higher than those in patients with late-onset PE or in normotensive controls, with similar values reported for the latter two groups (33). These findings provide molecular evidence that the production of placental misfolded proteins and placental ERS may contribute to early- but not to late-onset PE or normotensive controls. The present findings suggest that the rate of urinary congophilia in women with early-onset PE is significantly higher than that in the late-onset group and the normotensive group, indicating that the misfolded proteins in the urine may be linked to the pathogenesis of early-onset PE and the main source of misfolded proteins in the urine of patients with early-onset PE may be the placenta. In addition, our finding that the rate of urinary congophilia in the late-onset PE group is significantly higher than that in the normotensive group indicates that the main source of the misfolded proteins in the urine of patients with late-onset PE is different from that of the patients in the normotensive group, and probably not the placenta. Furthermore, previous studies have demonstrated the presence of the same types of misfolded proteins in both the plasma and urine of PE patients (21, 43). Accordingly, we propose that the urinary misfolded proteins in late-onset PE may be derived from the plasma.

Studies by Buhimschi et al. have demonstrated that the assessment of urinary congophilia with the Congo red dot test is useful for the prognostication of medically indicated delivery in patients with PE, suggesting a close link between urinary congophilia and PE cases with severe maternal and fetal complications (33). In addition, previous studies have demonstrated that placental or early-onset PE is associated with a high risk of maternal and fetal complications (6, 44, 45). In the present study, the rate of urinary congophilia in the early-onset PE group was significantly higher than that in the late-onset group, suggesting an association between urinary congophilia and early-onset PE. Nevertheless, the present findings are inconsistent with those of Nagarajappa, whereby the Congo red retention value was lower in the early- than in the late-onset PE group. Furthermore, findings of this study suggest that urinary congophilia is an independent risk factor for iatrogenic prematurity in early-onset PE patients; in fact, the present findings indicate that early-onset PE patients with urinary congophilia are at a higher risk of adverse pregnancy outcomes than are their counterparts without urinary congophilia. Based on the present findings, we propose that urinary congophilia, confirmed with the CapCord test, may support the prognostication of pregnancy outcomes in patients with early-onset PE. In other words, the urine samples of patients with early-onset PE could be collected at the time of disease onset to detect the congophilia with CapCord test, the patients with positive urinary congophilia are more likely to present adverse maternal and neonatal outcomes such as HELLP syndrome and iatrogenic prematurity, so the medically indicated delivery, as an effective treatment, should be expected as early as possible for those patients. Conversely, the patients with negative urinary congophilia probably present a relatively better maternal and neonatal outcome, so the supportive and expectant treatment should be preferred, and iatrogenic prematurity would be avoided as far as possible to extend the latency period between the onset and delivery.

In summary, results of this study suggest that the main sources of the urinary misfolded proteins in early- and late-onset PE seem different, the former may be the placenta, but the latter may be the plasma. To the best of our knowledge, this study is first to assess the association of urinary congophilia confirmed with the CapCord test with the pregnancy outcomes of Chinese patients with early-onset PE. Urinary congophilia confirmed with the CapCord test may be useful in the prognostication of pregnancy outcomes in patients with early-onset PE. However, this study has some limitations. First, the study population was composed of Chinese patients; thus, the conclusion of this study may not be applicable to other populations. Second, the sample size is relatively small, and the error of the CapCord test and influence of gestational age on urinary congophilia are not excluded in this study. Thus, more studies that consider the influence of gestational age on urinary congophilia with CapCord test are needed to validate the present findings.

## Data Availability Statement

The raw data supporting the conclusions of this article will be made available by the authors, without undue reservation.

## Ethics Statement

The studies involving human participants were reviewed and approved by Institutional Review Board of Shengjing Hospital of China Medical University. Written informed consent for participation was not required for this study in accordance with the national legislation and the institutional requirements.

## Author Contributions

BC: conceptualized the study and wrote the protocol and the manuscript. XY, XL, and JX: participated in the experiment and confirmed the experimental results. JD: interpreted the data and reviewed and edited the manuscript. All authors contributed to the article and approved the submitted version.

## Conflict of Interest

XY, XL, and JX are employed by Shuwen Biotech Company Ltd., China. The remaining authors declare that the research was conducted in the absence of any commercial or financial relationships that could be construed as a potential conflict of interest.

## Publisher's Note

All claims expressed in this article are solely those of the authors and do not necessarily represent those of their affiliated organizations, or those of the publisher, the editors and the reviewers. Any product that may be evaluated in this article, or claim that may be made by its manufacturer, is not guaranteed or endorsed by the publisher.

## References

[1] Duley L. The global impact of pre-eclampsia and eclampsia. Semin Perinatol. (2009) 33:130–7. 10.1053/j.semperi.2009.02.010 19464502

[2] Brown MA Magee LA Kenny LC Karumanchi SA McCarthy FP Saito S. Hypertensive disorders of pregnancy: ISSHP classification, diagnosis, and management recommendations for international practice. Hypertension. (2018) 72:24–43. 10.1161/HYPERTENSIONAHA.117.10803 29899139

[3] Wildman K Bouvier-Colle MH Group M. Maternal mortality as an indicator of obstetric care in Europe. BJOG. (2004) 111:164–9. 10.1046/j.1471-0528.2003.00034.x-i1 14723755

[4] Sircar M Thadhani R Karumanchi SA. Pathogenesis of preeclampsia. Curr Opin Nephrol Hypertens. (2015) 24:131–8. 10.1097/MNH.0000000000000105 25636145

[5] Ghulmiyyah L Sibai B. Maternal mortality from preeclampsia/eclampsia. Semin Perinatol. (2012) 36:56–9. 10.1053/j.semperi.2011.09.011 22280867

[6] von Dadelszen P Magee LA Roberts JM. Subclassification of preeclampsia. Hypertens Pregnancy. (2003) 22:143–8. 10.1081/PRG-120021060 12908998

[7] Odegard RA Vatten LJ Nilsen ST Salvesen KA Austgulen R. Risk factors and clinical manifestations of pre-eclampsia. BJOG. (2000) 107:1410–6. 10.1111/j.1471-0528.2000.tb11657.x 11117771

[8] Levine RJ Maynard SE Qian C Lim KH England LJ Yu KF. Circulating angiogenic factors and the risk of preeclampsia. N Engl J Med. (2004) 350:672–83. 10.1056/NEJMoa031884 14764923

[9] Masuyama H Segawa T Sumida Y Masumoto A Inoue S Akahori Y. Different profiles of circulating angiogenic factors and adipocytokines between early- and late-onset pre-eclampsia. BJOG. (2010) 117:314–20. 10.1111/j.1471-0528.2009.02453.x 20015306

[10] Gilbert JS Babcock SA Granger JP. Hypertension produced by reduced uterine perfusion in pregnant rats is associated with increased soluble fms-like tyrosine kinase-1 expression. Hypertension. (2007) 50:1142–7. 10.1161/HYPERTENSIONAHA.107.096594 17923588

[11] Makris A Thornton C Thompson J Thomson S Martin R Ogle R. Uteroplacental ischemia results in proteinuric hypertension and elevated sFLT-1. Kidney Int. (2007) 71:977–84. 10.1038/sj.ki.5002175 17377512

[12] Kaufmann P Black S Huppertz B. Endovascular trophoblast invasion: implications for the pathogenesis of intrauterine growth retardation and preeclampsia. Biol Reprod. (2003) 69:1–7. 10.1095/biolreprod.102.014977 12620937

[13] Aguzzi A Haass C. Games played by rogue proteins in prion disorders and Alzheimer's disease. Science. (2003) 302:814–8. 10.1126/science.1087348 14593165

[14] Soto C. Unfolding the role of protein misfolding in neurodegenerative diseases. Nat Rev Neurosci. (2003) 4:49–60. 10.1038/nrn1007 12511861

[15] Labbadia J Morimoto RI. The biology of proteostasis in aging and disease. Annu Rev Biochem. (2015) 84:435–64. 10.1146/annurev-biochem-060614-033955 25784053 PMC4539002

[16] Belay ED. Transmissible spongiform encephalopathies in humans. Annu Rev Microbiol. (1999) 53:283–314. 10.1146/annurev.micro.53.1.283 10547693

[17] Iwasaki Y. Creutzfeldt-Jakob disease. Neuropathology. (2017) 37:174–88. 10.1111/neup.12355 28028861

[18] Brown K Mastrianni JA. The prion diseases. J Geriatr Psychiatry Neurol. (2010) 23:277–98. 10.1177/0891988710383576 20938044

[19] Buhimschi IA Zhao G Funai EF Harris N Sasson IE Bernstein IM. Proteomic profiling of urine identifies specific fragments of SERPINA1 and albumin as biomarkers of preeclampsia. Am J Obstet Gynecol. (2008) 199:551 e1–16. 10.1016/j.ajog.2008.07.006 18984079 PMC2679897

[20] Millen KR Buhimschi CS Zhao G Rood KM Tabbah S Buhimschi IA. Serum and urine thioflavin-T-enhanced fluorescence in severe preeclampsia. Hypertension. (2018) 71:1185–92. 10.1161/HYPERTENSIONAHA.118.11034 29686018 PMC5945331

[21] Buhimschi IA Nayeri UA Zhao G Shook LL Pensalfini A Funai EF. Protein misfolding, congophilia, oligomerization, and defective amyloid processing in preeclampsia. Sci Transl Med. (2014) 6:245ra92. 10.1126/scitranslmed.3008808 25031267

[22] Tong M Cheng SB Chen Q DeSousa J Stone PR James JL. Aggregated transthyretin is specifically packaged into placental nano-vesicles in preeclampsia. Sci Rep. (2017) 7:6694. 10.1038/s41598-017-07017-x 28751735 PMC5532246

[23] Cater JH Kumita JR Zeineddine Abdallah R Zhao G Bernardo-Gancedo A Henry A. Human pregnancy zone protein stabilizes misfolded proteins including preeclampsia- and Alzheimer's-associated amyloid beta peptide. Proc Natl Acad Sci USA. (2019) 116:6101–10. 10.1073/pnas.1817298116 30850528 PMC6442606

[24] Cheng SB Nakashima A Sharma S. Understanding pre-eclampsia using Alzheimer's etiology: an intriguing viewpoint. Am J Reprod Immunol. (2016) 75:372–81. 10.1111/aji.12446 26585303

[25] Li XM Liu XM Xu J Du J Cuckle H. Late pregnancy screening for preeclampsia with a urinary point-of-care test for misfolded proteins. PLoS ONE. (2020) 15:e0233214. 10.1371/journal.pone.0233214 32433710 PMC7239432

[26] Ashburn TT Han H McGuinness BF Lansbury PT Jr. Amyloid probes based on congo red distinguish between fibrils comprising different peptides. Chem Biol. (1996) 3:351–8. 10.1016/S1074-5521(96)90118-0 8807864

[27] Stopa B Piekarska B Konieczny L Rybarska J Spolnik P Zemanek G. The structure and protein binding of amyloid-specific dye reagents. Acta Biochim Pol. (2003) 50:1213–27. 10.18388/abp.2003_3645 14740008

[28] Klunk WE Pettegrew JW Abraham DJ. Quantitative evaluation of congo red binding to amyloid-like proteins with a beta-pleated sheet conformation. J Histochem Cytochem. (1989) 37:1273–81. 10.1177/37.8.2666510 2666510

[29] Inouye H Kirschner DA. Alzheimer's beta-amyloid: insights into fibril formation and structure from congo red binding. Subcell Biochem. (2005) 38:203–24. 10.1007/0-387-23226-5_10 15709480

[30] Buell AK Dobson CM Knowles TP Welland ME. Interactions between amyloidophilic dyes and their relevance to studies of amyloid inhibitors. Biophys J. (2010) 99:3492–7. 10.1016/j.bpj.2010.08.074 21081099 PMC2980715

[31] Burton GJ Yung HW Cindrova-Davies T Charnock-Jones DS. Placental endoplasmic reticulum stress and oxidative stress in the pathophysiology of unexplained intrauterine growth restriction and early onset preeclampsia. Placenta. (2009) 30(Suppl. A):S43–8. 10.1016/j.placenta.2008.11.003 19081132 PMC2684656

[32] Mizuuchi M Cindrova-Davies T Olovsson M Charnock-Jones DS Burton GJ Yung HW. Placental endoplasmic reticulum stress negatively regulates transcription of placental growth factor via ATF4 and ATF6beta: implications for the pathophysiology of human pregnancy complications. J Pathol. (2016) 238:550–61. 10.1002/path.4678 26648175 PMC4784173

[33] Yung HW Atkinson D Campion-Smith T Olovsson M Charnock-Jones DS Burton GJ. Differential activation of placental unfolded protein response pathways implies heterogeneity in causation of early- and late-onset pre-eclampsia. J Pathol. (2014) 234:262–76. 10.1002/path.4394 24931423 PMC4277692

[34] Hetz C. The unfolded protein response: controlling cell fate decisions under ER stress and beyond. Nat Rev Mol Cell Biol. (2012) 13:89–102. 10.1038/nrm3270 22251901

[35] Kaufman RJ Scheuner D Schroder M Shen X Lee K Liu CY. The unfolded protein response in nutrient sensing and differentiation. Nat Rev Mol Cell Biol. (2002) 3:411–21. 10.1038/nrm829 12042763

[36] Zhang K Kaufman RJ. From endoplasmic-reticulum stress to the inflammatory response. Nature. (2008) 454:455–62. 10.1038/nature07203 18650916 PMC2727659

[37] McCarthy FP Adetoba A Gill C Bramham K Bertolaccini M Burton GJ. Urinary congophilia in women with hypertensive disorders of pregnancy and preexisting proteinuria or hypertension. Am J Obstet Gynecol. (2016) 215:464 e1–7. 10.1016/j.ajog.2016.04.041 27133010

[38] Nagarajappa C Rangappa SS Suryanarayana R Balakrishna S. Urinary congophilia in preeclampsia: experience from a rural tertiary-care hospital in India. Pregnancy Hypertens. (2018) 13:83–6. 10.1016/j.preghy.2018.05.006 30177078

[39] Rood KM Buhimschi CS Dible T Webster S Zhao G Samuels P. Congo red dot paper test for antenatal triage and rapid identification of preeclampsia. EClin Med. (2019) 8:47–56. 10.1016/j.eclinm.2019.02.004 31193633 PMC6537515

[40] Mao XR Crowder CM. Protein misfolding induces hypoxic preconditioning via a subset of the unfolded protein response machinery. Mol Cell Biol. (2010) 30:5033–42. 10.1128/MCB.00922-10 20733002 PMC2953055

[41] Paschen W Mengesdorf T. Endoplasmic reticulum stress response and neurodegeneration. Cell Calcium. (2005) 38:409–15. 10.1016/j.ceca.2005.06.019 16087231

[42] Jian B Hsieh CH Chen J Choudhry M Bland K Chaudry I. Activation of endoplasmic reticulum stress response following trauma-hemorrhage. Biochim Biophys Acta. (2008) 1782:621–6. 10.1016/j.bbadis.2008.08.007 18801427 PMC2628582

[43] Buhimschi I Zhao G Saade G Buhimschi CS. Evidence for alpha-1-antitrypsin (A1AT) polymerization in preeclampsia: a novel mechanism for endothelial cell injury. Am J Obstetr Gynecol. (2006) 195:S150. 10.1016/j.ajog.2006.10.526

[44] Mongraw-Chaffin ML Cirillo PM Cohn BA. Preeclampsia and cardiovascular disease death: prospective evidence from the child health and development studies cohort. Hypertension. (2010) 56:166–71. 10.1161/HYPERTENSIONAHA.110.150078 20516394 PMC3037281

[45] Paruk F Moodley J. Maternal and neonatal outcome in early- and late-onset pre-eclampsia. Semin Neonatol. (2000) 5:197–207. 10.1053/siny.2000.0023 10956445

